# Wide-Swath High-Resolution Immersed Grating Spectrometer for Greenhouse Gas Monitoring: Optical Design and Fabrication

**DOI:** 10.3390/s26134203

**Published:** 2026-07-03

**Authors:** Tuotuo Yang, Xinhua Chen, Qiao Pan, Zhicheng Zhao, Quan Liu, Weimin Shen

**Affiliations:** 1 School of Optoelectronic Science and Engineering, Soochow University, Suzhou 215006, China; 20234039002@stu.suda.edu.cn (T.Y.); panqiao@suda.edu.cn (Q.P.); zczhao@suda.edu.cn (Z.Z.); liuquan@suda.edu.cn (Q.L.); swm@suda.edu.cn (W.S.); 2 Key Lab of Advanced Optical Manufacturing Technologies of Jiangsu Province & Key Lab of Modern Optical Technologies of Education Ministry of China, Suzhou 215006, China

**Keywords:** greenhouse gas monitoring, wide swath, high spatial resolution, four-channel spectrometer, common slit, immersed grating, smile

## Abstract

**Highlights:**

**What are the main findings?**

- Through parameter analysis, rational optical layout design, and the design and fabrication of immersed gratings, the developed spectrometer achieves a 100 km swath width and a spatial resolution of 3 km × 3 km while meeting the required spectral resolution under constraints imposed by detector size, signal-to-noise ratio, and payload size and mass.
- A prism-based simultaneous correction method is proposed for smile and anamorphic beam compression induced by high-angular-dispersion immersed gratings. In addition, large-sized immersed gratings with high groove density are successfully fabricated using holographic exposure and ion-beam etching.

**What are the implications of the main findings?**

- A 100 km swath width improves coverage efficiency and shortens revisit time, while a spatial resolution of 3 km × 3 km enhances the capability for identifying localized emission sources. Their combination provides strong support for regional emission inversion, quantification of natural sources and sinks, and studies of key carbon-cycle processes.
- The proposed prism-based simultaneous correction method determines the configuration and initial parameters of the dispersion module, providing a good design starting point and avoiding the blindness of parameter selection. The development of large-sized immersed gratings with high groove density also provides technical support for future high-performance grating spectrometers.

**Abstract:**

Spaceborne spectrometers are key optical payloads for global and regional greenhouse gas (GHGs) monitoring. With the increasing demands for high-precision and high-efficiency monitoring, spectrometers are required to provide a wide swath, high spatial resolution, and high spectral resolution. However, existing spaceborne grating spectrometers still face a trade-off between swath width and spatial resolution. To address this issue, this paper presents the optical design and fabrication of an immersed-grating spectrometer for GHG monitoring. The proposed spectrometer achieves a swath width of 100 km and a spatial resolution of 3 km × 3 km while providing high spectral resolution. It operates in four channels centered at 0.76, 1.61, 2.06, and 2.30 μm, covering the O_2_-A band and the main absorption bands of CO_2_ and CH_4_, with corresponding spectral resolutions of 0.04, 0.07, 0.09, and 0.10 nm, respectively. The four channels share a common slit, which reduces system volume and inter-channel spatial registration errors. Immersed gratings are used as the core dispersive elements, enabling high spectral resolution in a compact optical configuration. To correct the smile and anamorphic beam compression induced by high-angular-dispersion immersed gratings, a prism-based simultaneous correction method is proposed. Based on this method, the initial parameters of the dispersion module are determined, and the optical design of the spectrometer is completed. Large-sized immersed gratings with high groove density are precisely fabricated using holographic lithography and ion-beam etching, after which the spectrometer is aligned and tested. The test MTF at the Nyquist frequency of the spatial dimension exceeds 0.72, indicating good imaging quality. The test spectral resolution of the four channels is all better than the design value, and the maximum smile and trapezoidal distortion are both within one pixel. This spectrometer provides an effective technical solution for achieving wide-swath, high-spatial-resolution, and high-spectral-resolution GHG monitoring under constraints imposed by detector size, signal-to-noise ratio, and payload size and mass.

## 1. Introduction

Greenhouse gases (GHGs), primarily carbon dioxide (CO_2_) and methane (CH_4_), have increased markedly since the Industrial Revolution, contributing substantially to anthropogenic climate change and posing major challenges to global ecosystems and human societies [1,2,3]. Accurate GHG monitoring is essential for identifying major emission sources, supporting emission reduction efforts, and improving the understanding of the global carbon cycle.

Ground-based GHG monitoring networks, such as the Total Carbon Column Observing Network (TCCON), provide highly accurate and stable measurements. However, their applications are limited by the uneven spatial distribution of stations and incomplete spatial coverage [4,5]. Satellite remote sensing has therefore become an important approach for global GHG monitoring because it provides wide coverage, near-real-time observations, and continuous long-term measurements [2,6]. Global satellite-based GHG monitoring began with the SCIAMACHY aboard ESA’s Envisat [7]. This instrument was the first to use shortwave-infrared absorption bands as detection channels. However, due to the limited spectral resolution and signal-to-noise ratio available at that time, its retrieval accuracy was only about 14 ppm, which could not satisfy quantitative inversion requirements. Later, Japan launched the GOSAT carrying the TANSO-FTS [8,9]. This instrument adopted Fourier-transform spectroscopy and achieved high spectral resolution over a broad spectral range, improving the CO_2_ column retrieval accuracy to approximately 1.5 ppm. Subsequently, several countries developed dedicated GHG spectrometers, such as the OCO-2 launched by the United States [10,11], the ACGS on China’s TanSat [1,12], and the GAS on FengYun-3D [13]. More recently, the TROPOMI on ESA’s Sentinel-5 Precursor [14,15] was the first to employ an immersed grating for shortwave-infrared observations, achieving an ultra-wide swath and significantly improving global coverage efficiency. These instruments can be broadly divided into two categories according to their dispersive principles: Fourier transform spectrometers (FTSs) and grating spectrometers. Although FTSs can acquire high-resolution spectra over a wide spectral range, they are generally limited by relatively low spatial resolution and sparse spatial sampling [16]. In contrast, grating spectrometers, such as OCO-2, ACGS, and TROPOMI, offer higher spatial resolution and enable continuous mapping through push-broom operation. For example, OCO-2 achieves a spatial resolution of 1.29 km × 2.25 km with a swath width of 10.6 km, whereas TROPOMI provides a much wider swath of 2600 km at a coarser spatial resolution of 7 km × 7 km.

Although existing grating-based spectrometers for GHG monitoring have enabled high-accuracy observations, they still face a trade-off between swath width and spatial resolution. OCO-2 and ACGS provide high spatial resolution, but their swath widths are limited, resulting in long revisit times. In contrast, TROPOMI achieves daily global coverage with a very wide swath, but its relatively low spatial resolution limits its ability to resolve fine-scale emission sources, such as individual facilities. This trade-off arises because the optical design of such spectrometers is simultaneously constrained by detector size, signal-to-noise ratio, spectral resolution requirements, and available payload resources.

Growing demands for global carbon budget accounting and precise emission-source identification have raised new requirements for spaceborne spectrometers. Future instruments are expected to simultaneously provide wide-swath coverage and high spatial resolution [17,18]. Wide-swath coverage helps shorten revisit time and improve observational efficiency, whereas high spatial resolution is essential for resolving emission sources in urban areas and even at the facility scale [19]. Their combination would provide stronger support for regional emission inversion, quantification of natural sources and sinks, and studies of key carbon-cycle processes [20,21]. This trend is also reflected in several next-generation missions. For example, GOSAT-GW is designed to achieve a spatial resolution of 3 km × 3 km with a swath width of 90 km [22,23,24], while CO2M, which also employs an immersed grating, targets a spatial resolution of 2 km × 2 km with a swath wider than 250 km [25,26].

To address the trade-off between swath width and spatial resolution in existing spaceborne grating spectrometers, this paper presents the optical design and fabrication of an immersed-grating spectrometer for global and regional GHGs observations. The optical system adopts a four-channel common-slit layout and employs immersed gratings as the core dispersive elements, thereby achieving a 100 km swath width, a spatial resolution of 3 km × 3 km, and high spectral resolution over the O_2_-A band and the main absorption bands of CO_2_ and CH_4_. In addition, a prism-based simultaneous correction method is proposed for smile and anamorphic beam compression induced by high-angular-dispersion immersed gratings, which enables the determination of the initial parameters of the dispersion module. The immersed gratings are manufactured using a holographic lithography–ion-beam etching process, and the spectrometer is subsequently fabricated, aligned, and tested. The results demonstrate that the proposed design provides an effective solution for achieving wide-swath, high-spatial-resolution, and high-spectral-resolution GHG monitoring under constraints imposed by detector size, signal-to-noise ratio, and payload size and mass.

The remainder of the paper is organized as follows: the parameters of the spectrometer are analyzed in Section 2. The optical design results and performance evaluation of the spectrometer are introduced in Section 3, with particular emphasis on the design method and initial parameter determination of the dispersion module. The fabrication of the spectrometer, along with the testing results and discussion, is presented in Section 4. The conclusions are presented in Section 5.

## 2. Materials and Methods: Parameter Analysis

In the monitoring of atmospheric CO_2_ and CH_4_ concentrations, solar radiation passes through the atmosphere, undergoes diffuse reflection at the Earth’s surface, travels back to space, and subsequently enters the spectrometer. Throughout this process, a fraction of the solar radiation is absorbed by gas molecules, resulting in the formation of characteristic spectral lines. The concentrations of these gas molecules can be determined by analyzing the depth of the absorption spectral lines [27,28].

To achieve precise monitoring of CO_2_ and CH_4_, the spectrometer employs four spectral channels with central wavelengths of 0.76 µm, 1.61 µm, 2.06 µm, and 2.3 µm, respectively. The 0.76 µm channel is designed to measure oxygen concentration, estimate surface pressure, and identify clouds, aerosols, and other atmospheric constituents. The 1.61 µm channel and the 2.06 µm channel are used for the measurement of CO_2_ and water vapor concentrations [11,28], while the 2.3 µm channel is specifically designed for the measurement of CH_4_ concentration. In the following text, channels B1 to B4 are used to denote these four spectral channels, respectively. We first analyze the spectral resolution of the spectrometer, which quantifies the instrument’s capability to resolve fine spectral features. Concurrently, this analysis enables the determination of the minimum signal-to-noise ratio (SNR) required to achieve the desired retrieval accuracy. Subsequently, the spectrometer’s F-number is established based on the required SNR. The analysis process described below is based on the B2 channel. For other channels, the analysis methods are similar. Finally, the primary optical parameters of the four channels of the spectrometer are presented.

### 2.1. Spectral Resolution and Minimum SNR Analysis

High spectral resolution is preferred to distinguish individual absorption spectral lines for GHG monitoring. However, this makes it more difficult to achieve a high SNR [21] and increases the complexity of the spectrometer. Therefore, it is necessary to determine a reasonable spectral resolution before the spectrometer design.

The Line-By-Line Radiative Transfer Model (LBLRTM) is an accurate and efficient model that resolves individual spectral lines for high-fidelity atmospheric radiative transfer. First, the spectral radiance *L*_0_(*λ*) at the entrance pupil is simulated using the LBLRTM version 12.8 [18]. The 1976 U.S. Standard Atmosphere model [29,30,31] is employed during the simulation, and the additional parameters utilized are listed in Table 1. Then, the spectral radiance *L*(*λ*) observed by the spectrometer can be calculated through the convolution of the spectral radiance *L*_0_(*λ*) at the pupil and the spectral response function (SRF), as expressed in Equation (1). The selected SRF is a Gaussian function, and its full width at half maximum (FWHM) is equal to the spectral resolution of the spectrometer.

(1) $$L\left(\lambda \right)=L_{0}\left(\lambda \right)⊗SRF\left(\lambda \right)$$

**Figure 1:**
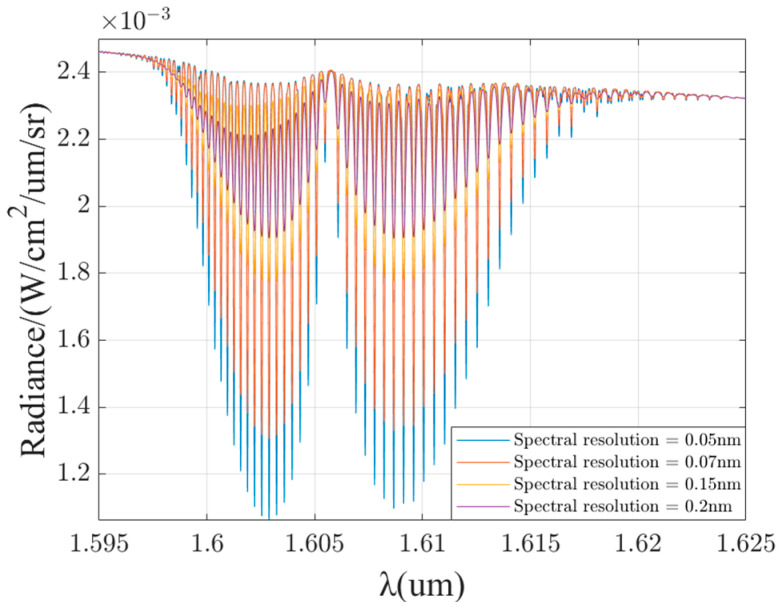
The simulated observed spectral radiance of 400 ppm CO_2_ concentration.

Based on the aforementioned simulation results, the curve of the spectral radiance observed by spectrometers under different spectral resolutions is presented in Figure 1. It can be found that this curve exhibits multiple distinct absorption peaks, and the spectral radiance of these absorption peaks decreases with increasing spectral resolution. At a spectral resolution of 0.2 nm, the minimum observed spectral radiance is 1.9 × 10^−3^ W/cm^2^/μm/sr, whereas it decreases to 1.3 × 10^−3^ W/cm^2^/μm/sr at a spectral resolution of 0.07 nm.

**Figure 2:**
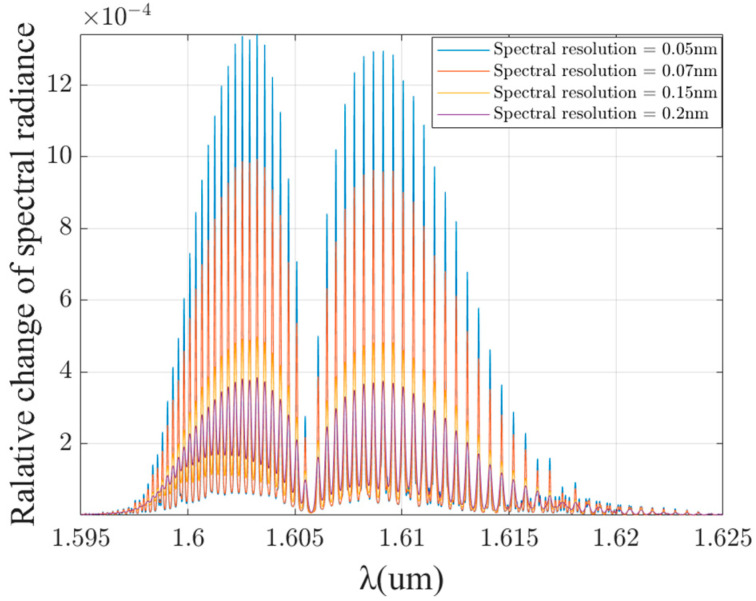
The relative radiation changes at different spectral resolutions.

The observed spectral radiance at the absorption peak wavelength varies with CO_2_ concentration. Therefore, the minimum signal-to-noise ratio (SNR) required to meet the retrieval accuracy can be determined by calculating the relative radiance change [19]. The relative radiance change is defined as the ratio of the radiance variation at an absorption peak induced by a 1 ppm concentration difference to the radiance value itself (ΔL/L). The spectral radiance under CO_2_ concentrations of 400 ppm and 401 ppm is calculated separately, and the corresponding relative radiance changes at different spectral resolutions are shown in Figure 2. The relative radiance change increases with improving spectral resolution. Taking a spectral resolution of 0.07 nm as an example, the peak relative radiance change caused by a 1 ppm CO_2_ variation is $9.92\times 10^{-4}$. To reliably detect this small change, the minimum SNR required for a single absorption line is approximately the reciprocal of this relative change [27]: ${\mathrm{SNR}}_{\mathrm{single}}=1/(9.92\times 10^{-4})\approx 1010$. If the spectral channel covers N independent absorption lines, the required SNR can be reduced by a factor of $1/\sqrt{N}$ through averaging [27]. For the B2 channel, 31 distinct absorption peaks are selected [30], i.e., N=31. Thus, the minimum SNR requirements at various spectral resolutions are summarized in Table 2. In general, higher spectral resolution reduces the required SNR, whereas lower spectral resolution causes information loss that must be compensated by a proportionally higher SNR. At a spectral resolution of 0.07 nm, a minimum SNR of 181 is required to achieve a CO_2_ concentration measurement accuracy of 1 ppm.

### 2.2. F-Number Determination and System Parameters

The F-number of the spectrometer is determined according to the required SNR, and the formula of the SNR is expressed as Equation (2) [18], where *S* is the measured signal, *N* is the noise, *L* is the spectral radiance at the entrance pupil of the spectrometer, τ is the transmittance of the optical system, *QE* is the detector quantum efficiency, *N_sr_* is the spectral sampling ratio, m is the number of pixel mergers in the spatial dimension, Δ*λ* is the detector pixel’s spectral bandwidth, *A_det_* is the detector pixel area, *F#* is the F number and *t* is the exposure time. The noise *N* consists of three terms: the shot noise $\sqrt{S}$, the detector dark signal noise $\sqrt{S_{dark}}$, and the electronic readout noise *N_read_*.

(2) $$SNR=\frac{S}{N}=\frac{L\times \tau \times QE\times \frac{A_{det}}{{F_{\#}}^{2}}\times m\times N_{\mathrm{sr}}\times \Delta \lambda \times t}{\sqrt{S+S_{dark}+N_{read}^{2}}}$$

**Figure 3:**
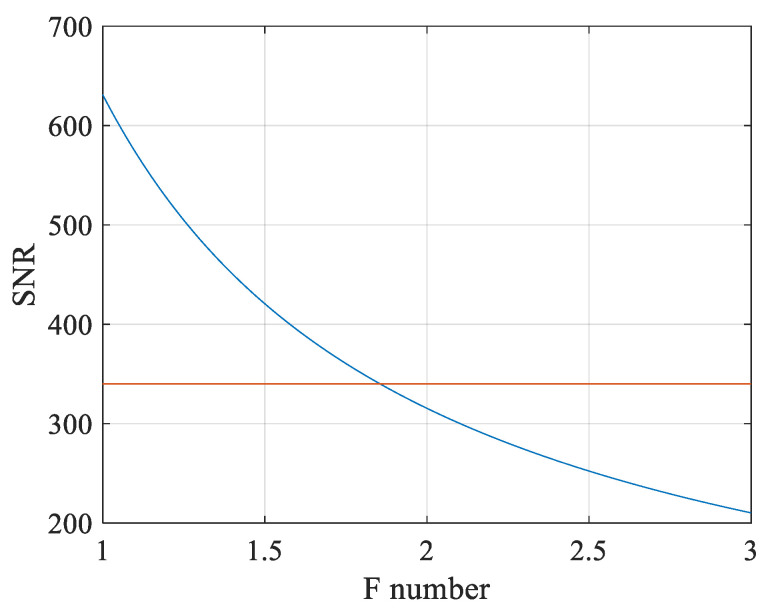
SNR as a function of F-number. (The red line indicates SNR = 340).

When *τ* = 0.48, *QE* = 0.8, *t* = 0.35 s, *m = 10*, *N_sr_* = 3, Δ*λ* = 0.023 nm, *A_det_* = 25 × 25 um^2^, *S_dark_* = 50 e^−^, *N_read_* = 160, the curve of SNR with different F-number is shown in Figure 3. Based on the minimum required SNR calculated in the previous section and a sufficient design margin, the minimum SNR for this channel is selected to be 340 [30]. Therefore, the F-number of this channel is determined to be 1.8 to fulfill the SNR requirement.

Based on the above analysis of spectral resolution and F-number, the optical parameters of the spectrometer with four channels are summarized in Table 3. The orbit altitude is 836 km, located on a sun-synchronous orbit. This orbit is commonly adopted by greenhouse gas monitoring satellites, such as TROPOMI (824 km) [14] and GOSAT-2 (613 km) [8]. The swath width is 100 km, which enables more frequent revisits and improved observation efficiency compared to narrow-swath missions such as OCO-2 [10,11] and ACGS [12]. The spatial resolution is 3 km, which is primarily intended for the identification and characterization of moderate-scale emission sources, including urban clusters, large power plants, and industrial facilities [6,19,22]. The combination of a 100 km swath width and a 3 km spatial resolution is comparable to that of GOSAT-GW, reflecting the trend among GHG monitoring missions to balance wide swath width and high spatial resolution. From the orbital altitude and swath width, the field of view is derived as 7°. The focal length of the spectrometer is calculated according to Equation (3). where GSD is the ground sampling distance (equal to the spatial resolution), and p_eff_ is the effective pixel size in spatial dimension.

(3) $$f=p_{eff}\cdot \frac{H}{GSD}$$

The resulting focal lengths are listed in Table 3.

Table 4 compares the key parameters of the spectrometer developed in this work with those of currently operational and under-development grating spectrometers for GHG monitoring. In terms of dispersive elements, the launched mission TROPOMI uses an immersed grating, whereas OCO-2 and ACGS employ plane gratings. Among next-generation instruments, CO2M and the spectrometer developed in this work both employ immersed gratings, highlighting the advantages of immersed gratings for achieving high spectral resolution in a compact system. With respect to spatial resolution and swath width, OCO-2 and ACGS achieve high spatial resolutions of 1–2 km but with relatively narrow swaths of 10–18 km, whereas TROPOMI provides a swath of 2600 km at a coarser spatial resolution of 7 km. Next-generation missions such as GOSAT-GW and CO2M are designed to pursue both wide swath and high spatial resolution. The spectrometer developed in this work achieves a swath width of 100 km and a spatial resolution of 3 km, which is comparable to missions such as GOSAT-GW in this respect. In addition, this spectrometer adopts a four-channel configuration. Compared with the three-channel configuration of OCO-2, ACGS, and CO2M, it covers the O_2_-A band (0.76 μm), the weak CO_2_ band (1.61 μm), and the strong CO_2_ band (2.06 μm), while additionally including a dedicated CH_4_ absorption band at 2.3 μm. The spectral resolutions of the four channels are 0.04, 0.07, 0.09, and 0.10 nm, respectively, which are comparable to those of OCO-2 and ACGS and finer than TROPOMI’s spectral resolutions in the SWIR bands. Overall, based on the comparison in Table 4, the spectrometer developed in this work demonstrates competitive performance relative to representative next-generation international missions in terms of swath width, spatial resolution, and spectral resolution.

## 3. Materials and Methods: Optical Design and Analysis

**Figure 4:**
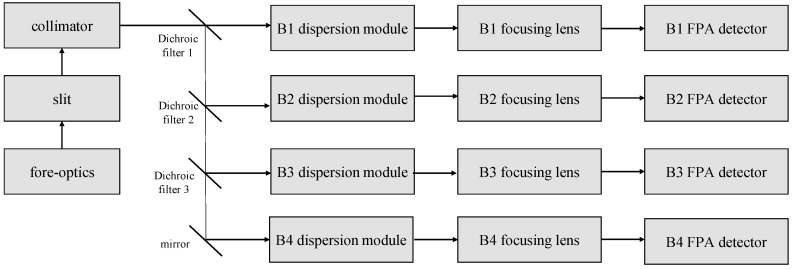
Layout block diagram of the spectrometer.

For a spectrometer with multiple channels, the spatial misregistration among the channels limits the instrument’s ability to quantitatively characterize GHGs. To avoid this issue, the spectrometer designed in this paper shares a common aperture and the slit, which not only enables a compact design with reduced size but also reduces the alignment difficulty between channels. Based on this design concept, the layout block diagram of the spectrometer is shown in Figure 4. The spectrometer is composed of fore-optics, slit, collimator, dichroic filters, dispersion modules, and focusing lenses. The fore-optics, slit, and collimator are shared by the four spectral channels. The incident light from the entrance of the spectrometer is focused onto the slit by the fore-optics, and after passing through the slit, it is collimated by the following collimator. Subsequently, the collimated light is divided into four spectral channels by the dichroic filters and directed into the corresponding dispersion modules. After dispersion, the light is focused by the focusing lenses and finally detected by the focal plane array (FPA) detector.

This section presents the design of the spectrometer’s fore-optics, collimator, dispersion modules, and focusing lens. The design and optimization of the dispersion modules are especially emphasized, for it determines the final performance of the spectrometer.

### 3.1. Fore-Optics and Collimator

The spectrometer features a spectral range covering the near-infrared to short-infrared wavelengths, and therefore, a reflective system is more suitable for the fore-optics and collimator due to its inherent achromatic advantage. In the design of the fore-optics, a real entrance pupil is necessary to reduce the physical dimensions of the pointing mirror and polarization scrambler. Meanwhile, the collimator must incorporate a real exit pupil with a large exit pupil distance, as this location is ideally suited for the immersed grating of the subsequent detection module. During the design process, the focal length ratio between the collimator and the fore-optics is carefully selected. A large focal length ratio may lead to an oversized grating, while a small ratio increases the required grating groove density to meet the required spectral resolving power.

**Figure 5:**
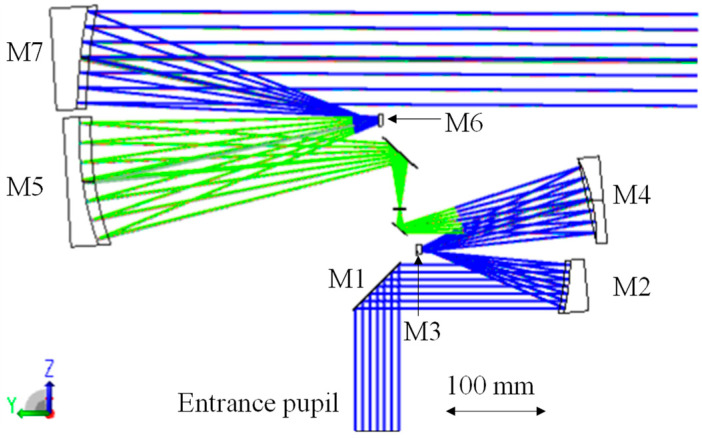
The fore-optics and collimator system optical layout.

Both fore-optics and collimator employ a Korsch type three-mirror-anastigmat (TMA) optical system [32,33]. The optical layout is shown in Figure 5, where M1 is a plane mirror, M3 and M6 are spherical mirrors, while M2, M4, and M5 are ellipsoidal mirrors. The entrance pupil diameter of the fore-optics is 45 mm with an FOV of 7°, and the exit pupil diameter of the collimator is 100 mm with an FOV of 3.15°.

### 3.2. Dispersion Module

The dispersion module is the core part of the spectrometer, directly determining the spectral performance. To achieve the high spectral resolution required for GHG monitoring, the Littrow configuration is preferred. In order to enhance dispersion capacity and improve compactness, the immersed grating, rather than a conventional plane grating, is used in this configuration [34].

The angular dispersion of the immersed grating directly determines the spectral resolution of the spectrometer and is a core parameter of the spectrometer. However, while achieving high dispersion, the immersed grating will introduce problems such as smile and anamorphic beam compression. Among them, smile causes spectral aliasing at the image plane and is the main aberration affecting the spectral quality [35,36]. Anamorphic beam compression increases the F number in the dispersion direction of the optical system. The increase in F number broadens the point spread function in the spectral dimension, reducing spectral resolution and also lowering the signal-to-noise ratio of the spectrometer. Ultimately, this affects the monitoring accuracy of GHGs. Therefore, the smile and anamorphic beam compression introduced by the immersed grating must be corrected during the design stage. In this section, the immersed grating is first analyzed theoretically, then the initial parameters of the dispersion module are determined, and finally the design results are presented. The design methods of the dispersion modules for the four channels are similar. Therefore, this section presents the design and results using the B2 channel as a representative example.

#### 3.2.1. Theoretical Analysis of Immersed Gratings

1. Angular Dispersion of the Immersed Grating

**Figure 6:**
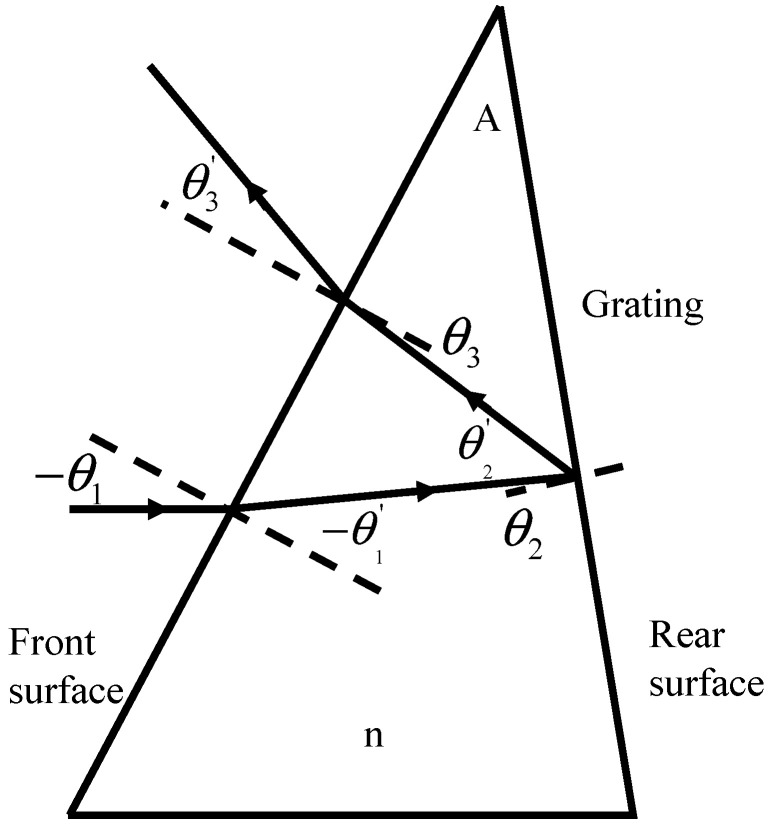
The schematic diagram of the immersed grating.

The schematic diagram of the immersed grating is shown in Figure 6. The front surface of the immersed grating is a refractive surface, and the rear surface is a reflective grating. The incident light is first refracted at the front surface, subsequently propagates toward the rear surface, and returns to the front surface after diffraction. A is the apex angle of the immersed grating, and n is the refractive index of the immersed medium. *θ_i_* (*i* = 1–3) represents the incident angle of each surface, and *θ’_i_* (*i* = 1–3) represents the emergent angle of each surface. Unlike traditional planar gratings, the grating surface of the immersed grating is immersed in a medium. The incident light and the diffracted light on the grating surface are both propagating in the immersed medium [37]. This will cause the coherent light beams of the grating diffraction to have a greater optical path difference, thereby achieving a higher resolving power.

During the calculation process, the sign conventions for these angles are defined as follows: the angle is positive when the ray rotates counterclockwise relative to the normal vector, and negative when the rotation is clockwise.

The incident light is first refracted at the front surface and then reaches the rear surface, where diffraction occurs. According to the grating equation in Equation (4) [37], the diffraction angle *θ’*_2_ is expressed as Equation (5), where *θ*_2_ is the incident angle on the grating, *d* is the grating period, *m* is the diffraction order, and *λ* is the wavelength.

(4) $$nd(\sin\theta_{2}+\sin\theta_{2}^{\prime })=m\lambda$$

(5) $$\sin\theta_{2}^{\prime }=m\lambda /nd-\sin\theta_{2}$$

The angular dispersion of the grating surface is then given by Equation (6).

(6) $$\frac{d\theta_{2}^{\prime }}{d\lambda }=\frac{m}{nd\cos\theta_{2}^{\prime }}$$

Upon returning to the front surface, the incident angle *θ*_3_ and its corresponding angular dispersion are given by Equations (7) and (8), respectively.

(7) $$\theta_{3}=\theta_{2}^{\prime }-A$$

(8) $$\frac{d\theta_{3}}{d\lambda }=\frac{d\theta_{2}^{\prime }}{d\lambda }$$

After refraction at the front surface, the refracted angle *θ’*_3_ and its angular dispersion are described by Equations (9) and (10), respectively.

(9) $$\sin\theta_{3}^{\prime }=n\sin\theta_{3}$$

(10) $$\frac{d\theta_{3}^{\prime }}{d\lambda }=n\frac{\cos\theta_{3}}{\cos\theta_{3}^{\prime }}\frac{d\theta_{3}}{d\lambda }$$

Finally, substituting Equations (6) and (8) into Equation (10) yields the angular dispersion of the immersed grating, as shown in Equation (11) [34].

(11) $$\frac{d\theta_{3}^{\prime }}{d\lambda }=\frac{m}{nd\cos\theta_{2}^{\prime }}\cdot n\frac{\cos\theta_{3}}{\cos\theta_{3}^{\prime }}$$

Equation (11) shows that the angular dispersion of the immersed grating is the product of two factors: the grating dispersion (first factor) and the dispersion induced by refraction (second factor). A refractive magnification factor k is introduced, as defined in Equation (12).

(12) $$k=\frac{\cos\theta_{3}}{\cos\theta_{3}^{\prime }}$$

**Figure 7:**
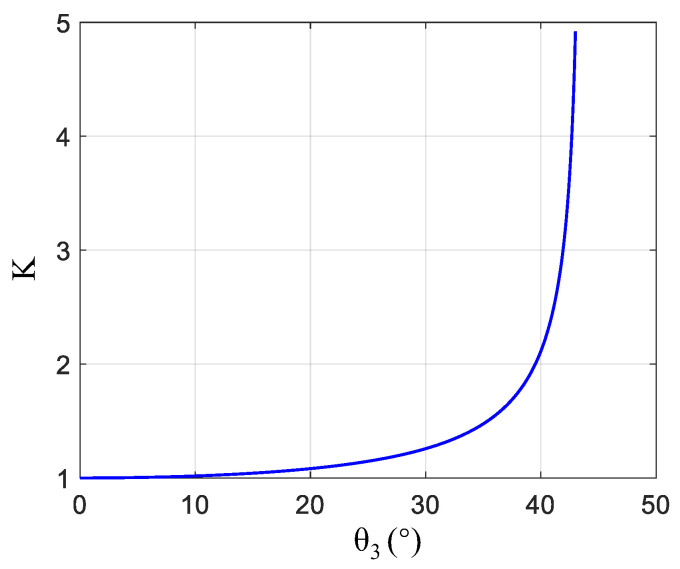
Variation in the magnification factor k with the incidence angle.

The magnification factor k varies with the incident angle *θ*_3_ as shown in Figure 7. The magnification factor k increases with *θ*_3_. The closer to the critical angle, the more obvious the change.

According to the spectral resolution requirements of the spectrometer, the immersed grating needs to have high angular dispersion. This can be achieved by reducing the grating period d of the immersed grating, which will simultaneously increase the diffraction angle *θ*′_2_ on the grating surface and enhance the dispersion capacity of the grating surface. Additionally, by increasing the magnification factor *K* = cosθ_3_/cos*θ*′_3_, the overall angular dispersion can be further improved.

2. Smile induced by Immersed Grating

**Figure 8:**
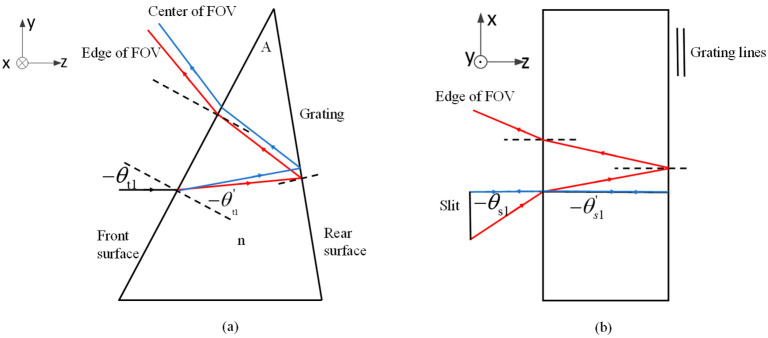
The principal section (**a**) and the sagittal section (**b**) of the immersed grating.

The smile induced by the immersed grating can be analyzed using the spatial ray tracing method. Figure 8a illustrates the projections of the chief rays onto the principal section of the immersed grating. Figure 8b depicts the projections of the chief rays on the sagittal section, and the grating line is oriented parallel to the slit. The blue and red lines represent the chief rays of the center and edge FOV, respectively. The polar angle is defined as the projection angle of the chief ray onto the principal section, whereas the azimuth angle refers to the projection angle onto the sagittal section. *θ_ti_* (*i =* 1–3) represents the incident polar angle of each surface, and *θ*′*_t__i_* (*i =* 1–3) represents the emergent polar angle of each surface. *θ_si_* (*i =* 1–3) represents the incident azimuth angle of each surface, and θ′_si_ (*i* = 1–3) represents the emergent azimuth angle of each surface.

By separately calculating the polar angle and azimuth angle of the principal ray after passing through each optical surface, the expression of the smile induced by the immersed grating can be obtained.

At the refractive surface, taking the front surface as an example, the calculation of the polar angle and the azimuth angle, respectively, follows the refraction law as shown in Equations (13) and (14).

(13) $$\sin\theta_{t1}^{\prime }=\sin\theta_{t1}/N$$

(14) $$\sin\theta_{s1}^{\prime }=\sin\theta_{s1}/n$$

where *N* is as shown in Equation (15) [36,38].

(15) $$N=\sqrt{n^{2}+(n^{2}-1)\cdot {\left(\tan\theta_{s1}\right)}^{2}}$$

On the grating surface, the incident polar angle and the diffraction polar angle follow Equation (16), where d represents the grating period. The azimuth angle follows Equation (17).

(16) $$n\cdot d\cdot \left(\sin\theta_{t2}+\sin\theta_{t2}^{\prime }\right)\cdot \cos\theta_{s2}=m\cdot \lambda$$

(17) $$\theta_{s2}^{\prime }=-\theta_{s2}$$

Finally, the polar angles of the exit chief rays for the center and edge FOV, denoted as *θ*′*_t_Center_* and *θ*′*_t_Edge_*, are given by Equations (18) and (19), respectively.

(18) $$\sin\theta_{t\_C\mathrm{enter}}^{\prime }=n\sin\left(\arcsin\left(\frac{m\lambda }{nd}-\sin\left(A+\arcsin\left(\frac{\sin\theta_{t1}}{n}\right)\right)\right)-A\right)$$

(19) $$\sin\theta_{t\_E\mathrm{dge}}^{\prime }=N\sin\left(\arcsin\left(\frac{m\lambda }{nd\cos\theta_{s2}}-\sin\left(A+\arcsin\left(\frac{\sin\theta_{t1}}{N}\right)\right)\right)-A\right)$$

It can be seen from Equations (18) and (19) that the polar angles of the exit chief rays in the central FOV and edge FOV are different, which leads to image centroid deviation in the spectral dimension on the image plane after passing through the focusing lens. This deviation is referred to as a smile, and its expression is given by Equation (20). Where *f_focus_* represents the focal length of the focusing lens.

(20) $$smile=f_{focus}\cdot \left(\theta_{t\_Edge}^{\prime }-\theta_{t\_Center}^{\prime }\right)$$

It can be found that Equations (18)–(20) are related to the grating parameters *m* and *d*, as well as the immersed medium parameters *A* and *n*. This indicates that the smile caused by the immersed grating is the result of the combined effect of the grating and the immersed medium. To achieve high angular dispersion, a smaller grating period is required for the immersed grating. At this time, the smile induced by the grating surface is dominant, while the refractive index of the immersed medium (silica) is relatively low, resulting in a smaller smile. Therefore, the silica immersed grating as a whole will have a relatively large smile, and prisms need to be introduced additionally for correction.

3. Anamorphic beam compression of the immersed grating

Anamorphic beam compression [39] of the immersed grating is characterized by the lateral magnification, defined as the ratio of the beam apertures in the dispersion direction. The lateral magnification of the immersed grating is expressed as shown in Equation (21), *D_1_* and *D_2_* denote the apertures of the incident and emergent beams, respectively.

(21) $$T=\frac{D_{2}}{D_{1}}=\frac{\cos\theta_{3}^{\prime }\cdot \cos\theta_{2}^{\prime }\cdot \cos\theta_{1}^{\prime }}{\cos\theta_{3}\cdot \cos\theta_{2}\cdot \cos\theta_{1}}$$

The lateral magnification is determined by the cosine ratios at three surfaces: the incident surface, the grating surface, and the exit surface. To achieve high angular dispersion, the immersed grating is typically designed with the diffraction angle at the grating surface being greater than the incident angle. In this case, the magnification factor at the grating surface satisfies cos*θ*′_2_/cosθ_2_ < 1, resulting in compression of the light beam. At the exit surface, when light emerges from the immersed medium into the air, compression also occurs, that is, cos*θ*′_3_/cos*θ*_3_ < 1. Additionally, increasing *k* = cosθ_3_/cos*θ*′_3_ can enhance the overall dispersion of the immersed grating, but it will inevitably lead to a further increase in the compression effect at the exit surface (cos*θ*′_3_/cos*θ*_3_). Therefore, to achieve a high angular dispersion, both the grating surface and the exit surface cause the beam aperture to be compressed, resulting in a total lateral magnification *T* < 1. Therefore, additional prisms are needed for correction.

#### 3.2.2. Optical Design of Dispersion Module

This section presents the optical design procedure of the dispersion module. To simultaneously satisfy the angular-dispersion requirement and correct the smile and anamorphic beam compression introduced by the immersed grating, a prism-based simultaneous correction method is adopted. First, the initial parameters of the immersed grating are determined according to the angular-dispersion requirement. Then, the prism parameters are selected to compensate for the smile and lateral magnification. Finally, the optical design results of the dispersion module are presented.

1. Prism-based Simultaneous Correction Method and Parameter Determination

The method for determining the initial parameters of the immersed grating is as follows.

An immersed grating operating under the Littrow condition achieves the high diffraction efficiency [40]. However, in this configuration the incident and diffracted beams are exactly superimposed, making it impossible to avoid mechanical interference with the optical components before and after the grating. Therefore, the immersed grating is actually operated in a quasi-Littrow configuration, where the diffraction angle is set larger than the incident angle, as shown in Equation (22), to maintain relatively high efficiency while obtaining sufficient spatial separation of the beams.

(22) $$\Delta \theta =\theta_{2}^{\prime }-\theta_{2}$$

**Figure 9:**
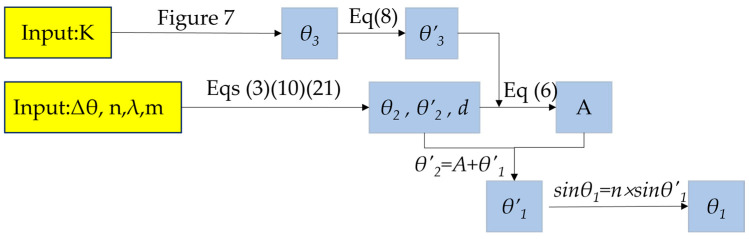
Calculation process of initial parameters for immersed gratings.

Once the amplification factor K and the angular separation Δθ are determined, the initial parameters of the immersed grating—the incidence angle $\theta_{1}$, the apex angle A, and the groove density—can be obtained using the calculation procedure shown in Figure 9. The specific steps are as follows. First, the value of $\theta_{3}$ corresponding to the chosen K is read from Figure 7, and $\theta_{3}^{\prime }$ is then calculated using Equation (9). Next, the grating equation (Equation (4)), the angular dispersion formula (Equation (11)), and Equation (22) are solved simultaneously to obtain $\theta_{2}$, $\theta_{2}^{\prime }$, and the grating period *d*. The apex angle A is then derived from Equation (7). Afterwards, $\theta_{1}^{\prime }$ is obtained from the geometric relation $\theta_{2}^{\prime }=A+\theta_{1}^{\prime }$. Finally, the incidence angle $\theta_{1}$ in air is determined using Snell’s law: $sin\theta_{1}=nsin\theta_{1}^{\prime }$.

A reasonable initial value of Δ*θ* needs to be selected. If Δ*θ* is too small, the spatial separation is insufficient; if Δ*θ* is too large, the grating deviates too far from the Littrow condition, and the diffraction efficiency drops significantly. After balancing engineering feasibility and diffraction efficiency, this paper selects Δ*θ* = 15° as the initial parameter.

The influence of the K value on the characteristics of the immersed grating is analyzed below. Theoretically, from Equation (11), when $\theta_{3}$ increases from 0 to the critical angle, K ranges from 1 to +∞. When K>2, according to the analysis in Section 3.2.1, the lateral magnification of the immersed grating becomes less than 0.5. This would require the beam diameter in the meridional direction incident on the immersed grating to be expanded by more than a factor of two, leading to an excessively large grating size and being detrimental to the miniaturization of the instrument. Therefore, the analysis is carried out for K values ranging from 1 to 2, which satisfies the practical engineering interval.

**Figure 10:**
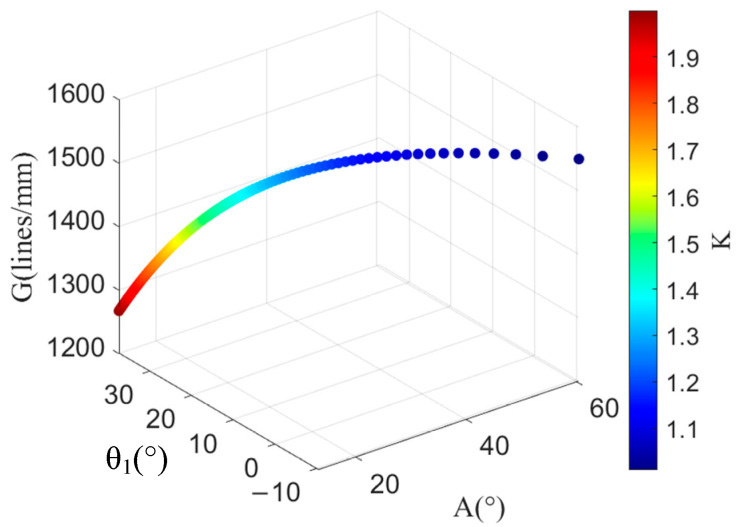
The parameter solutions of the immersed grating corresponding to K values of 1–2.

According to the calculation procedure shown in Figure 9, the parameter solutions of the immersed grating (incidence angle, apex angle, and groove density) corresponding to different K values are obtained. For K ranging from 1 to 2, the results are shown in Figure 10. The groove density varies from 1230 lines/mm to 1550 lines/mm, and it decreases as K increases.

**Figure 11:**
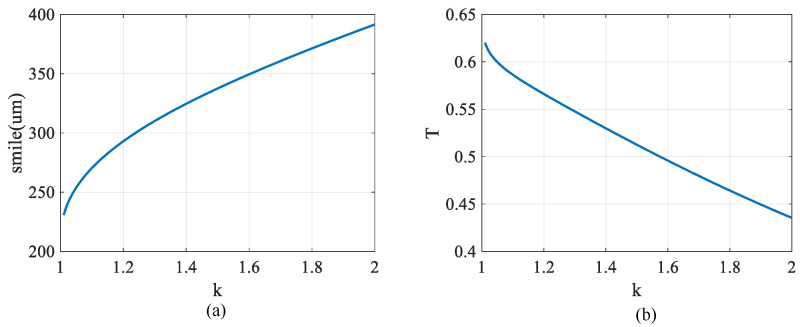
(**a**) The smile and (**b**) T variation curves with K caused by the immersion grating.

Substituting the initial parameters of the immersed grating corresponding to each K value into Equation (20) yields the smile of the immersed grating. The variation in smile with K is shown in Figure 11a. The smile ranges from 230 μm to 390 μm and increases with increasing K. Substituting the initial parameters of the immersed grating corresponding to each K value into Equation (21) yields the lateral magnification T of the immersed grating. The results are presented in Figure 11b. T ranges from 0.43 to 0.62 and decreases as K increases.

Considering the trade-off among groove density, smile, and lateral magnification T: when K is too small (e.g., K<1.2), the groove density exceeds 1500 lp/mm, making fabrication more difficult; when K is too large (e.g., K>1.6), T<0.5 leading to severe beam compression, and the smile exceeds 350 μm, increasing the difficulty of subsequent prism correction. Therefore, K values in the range of 1.2 to 1.6 can be regarded as a reasonable design interval. In this work, the intermediate value K=1.4 is selected as the initial design point, which yields a smile of about 324 μm and T=0.53. According to Figure 10, the corresponding initial parameters of the immersed grating are incidence angle 27.90°, apex angle 27.60°, and groove density 1438 lines/mm. This set of parameters serves as the initial input for subsequent Zemax OpticStudio 2023 optical optimization, and after further refinement all performance specifications are satisfied.

After determining the immersed-grating parameters, the prism parameters are selected to compensate for the induced smile and lateral magnification. To compensate for the lateral magnification of the immersed grating, the prisms are required to provide a lateral magnification of approximately 2-fold; meanwhile, to correct the smile, it is required to generate a smile with equal magnitude but opposite sign to that induced by the immersed grating.

Using the same spatial ray-tracing method, the smile expressions induced by the transmissive prism and the reflective prism can be obtained as given in Equations (23) and (24).

(23) $$smile_{t}=f_{focus}\cdot \left(N\cdot \sin\left(A-\arcsin\left(\sin\theta_{t1}/N\right)\right)-n\cdot \sin\left(A-\arcsin\left(\sin\theta_{t1}/n\right)\right)\right)$$

(24) $$smile_{r}=f_{focus}\cdot \left(N\cdot \sin\left(2A-\arcsin\left(\sin\theta_{t1}/N\right)\right)-n\cdot \sin\left(2A-\arcsin\left(\sin\theta_{t1}/n\right)\right)\right)$$

Analysis shows that the smile produced by a prism is always positive and oriented in the short-wavelength dispersion direction. Under the same incident angle and apex angle, a reflective prism induces a larger smile than a transmissive prism. In addition, the reflective prism can redirect the optical path, which is more conducive to reducing system volume. Therefore, the reflective prism is selected as the correction element in this design.

However, a single reflective prism cannot simultaneously provide the required twofold lateral magnification and compensate for the 324 μm smile induced by the immersed grating. Therefore, two reflective prisms are employed before the immersed grating. Together, these prisms provide the required twofold magnification, with each prism contributing a magnification factor of 1.414, while also compensating for part of the smile.

The lateral magnification of the reflective prism is given in Equation (25). Based on the magnification requirement and the need to avoid beam overlap, with a fixed 20° difference between the incident and emergent angles, the incident angle and apex angle of the reflective prism are determined to be 54.2° and 28.6°, respectively. Substituting these parameters into Equation (24) yields a smile of approximately 110 μm for each prism. Therefore, the two prisms together generate about 220 μm of smile, compensating for most of the smile introduced by the immersed grating. The residual smile is then corrected by a transmissive prism placed after the immersed grating.

(25) $$T=\frac{D_{2}}{D_{1}}=\frac{\cos\theta_{3}^{\prime }\cdot \cos\theta_{1}^{\prime }}{\cos\theta_{3}\cdot \cos\theta_{1}}$$

At this point, the initial parameters of the dispersion module have been determined. Accordingly, the prism-based simultaneous correction method adopted here consists of two reflective prisms placed before the immersed grating and one transmissive prism placed after it. The parameters of the key components are as follows: the two reflective prisms placed before the immersed grating have an incident angle of 54.2° and an apex angle of 28.6°, while the immersed grating has an incident angle of 29.32°, an apex angle of 25.9°, and a groove density of 1424 lines/m.

2. Optical Design of Dispersion Module

**Figure 12:**
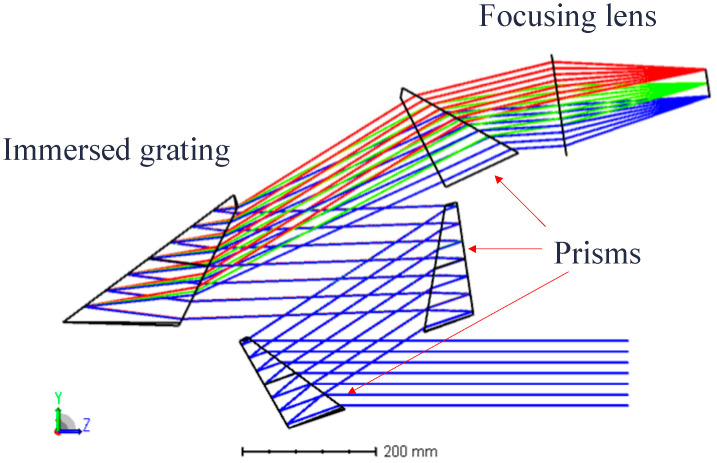
The optical layout of the B2 channel.

**Figure 13:**
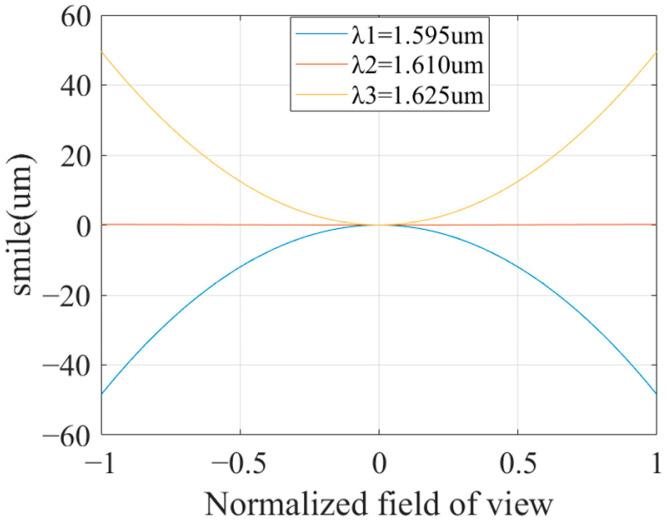
Smile of the B2 channel.

Based on the configuration and initial parameters of the dispersion module described above, the optical design of the dispersion module was completed through further optimization in Zemax. The optical layout of the B2 spectral channel is shown in Figure 12. The focusing lens is replaced by a paraxial surface with a focal length of 167 mm. The immersed grating operates under quasi-Littrow conditions, generating a smile that curves toward the apex angle. This smile is effectively compensated by the three prisms, which introduce an opposing smile that curves toward the bottom. As shown in Figure 13, the smile of the central wavelength is eliminated, while the smile of the edge wavelengths is about 54 µm, which can be corrected by the subsequent focusing lens.

These results indicate that the proposed prism-based simultaneous correction scheme can effectively suppress the smile introduced by the immersed grating and compensate for the lateral magnification while maintaining the required dispersion capability, thereby providing a feasible initial configuration for the subsequent optical design of the spectrometer.

### 3.3. Focusing Lens

**Figure 14:**
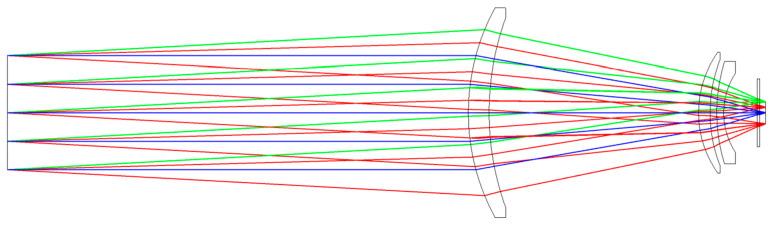
Optical layout of the B2 focusing lens.

The focusing lens features a large relative aperture; therefore, the Petzval configuration is preferred. This design consists of two lens groups with positive power. As shown in Figure 14, it is the focusing lens of Channel B2. The front group is a silicon lens, the rear surface of which is an even-order aspheric surface. The rear group comprises two lenses: a positive-power silicon lens and a negative-power fused silica lens. The combination of these two materials effectively eliminates the system’s chromatic aberration.

### 3.4. Optical System of Spectrometer and Performance Evaluation

**Figure 15:**
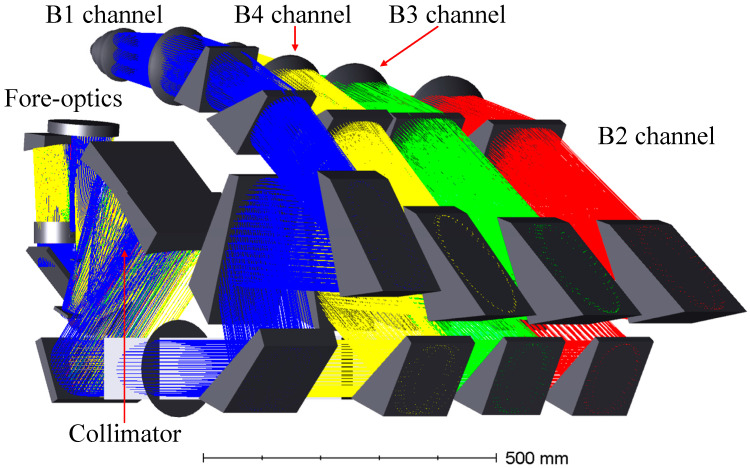
The optical layout of the spectrometer.

**Figure 16:**
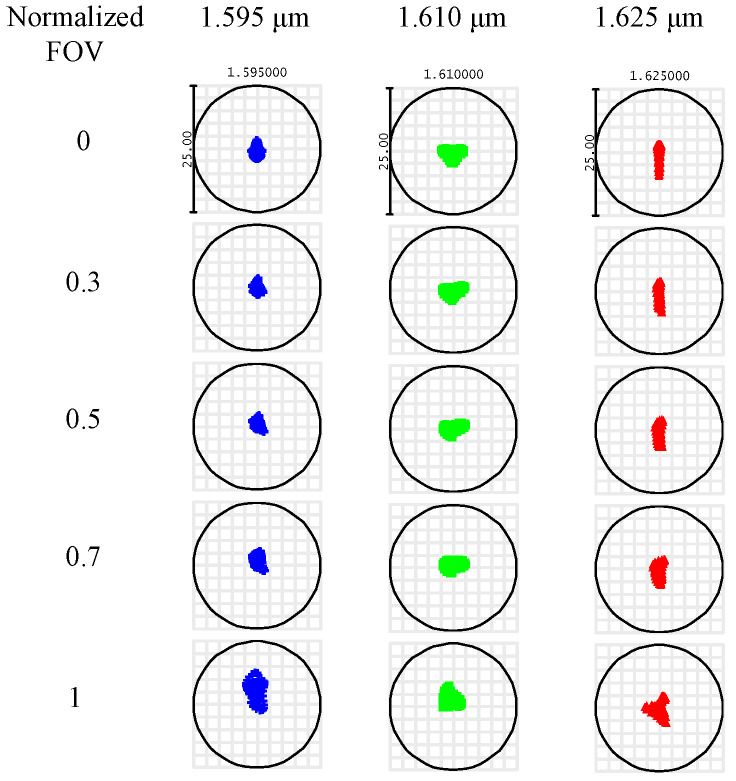
Spot diagrams of the B2 channel.

**Figure 17:**
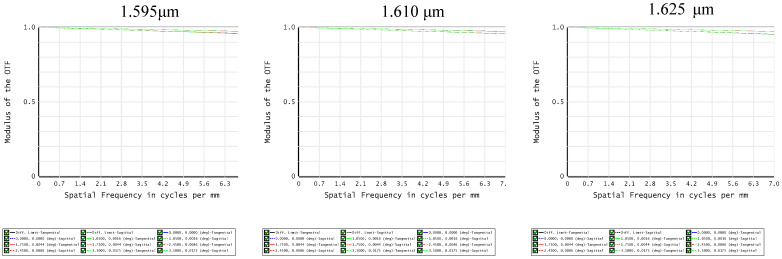
MTF curves for different wavelength.

**Figure 18:**
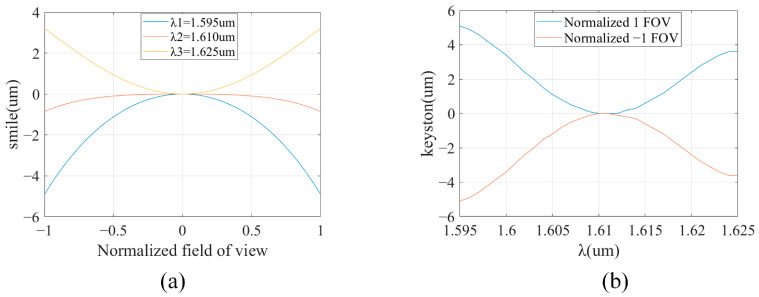
(**a**) Smile of the B2 channel; (**b**) keystone of the B2 channel.

The final optical layout of the spectrometer after optimization is shown in Figure 15. In this figure, the blue ray represents the light path of channel B1, the red ray represents B2, the green ray represents B3, and the yellow ray represents B4. The spot diagrams and MTF (Modulation Transfer Function) are used to evaluate the imaging quality of the spectrometer, while smile and keystone are analyzed to assess spectral performance. Take channel B2 as an example, the spot diagrams for different FOV and wavelength are shown in Figure 16. The maximum RMS radius of the spots is 4.2 µm, which is significantly smaller than the pixel size of 25 µm. Figure 17 shows the MTF curves at different wavelengths, and the MTF is higher than 0.95 at 7 lp/mm. Figure 18 shows the smile and keystone curves of the B2 channel. The smile of the central wavelength is 0.86 µm, while those at the edge wavelengths are 3.2 µm and 4.9 µm, respectively. The smile and keystone of the four channels of the spectrometer are shown in Table 5. The maximum smile is 8.1 µm, and the minimum value is 0.6 µm; the maximum value of keystone is 4.9 µm, and the minimum value is 0.2 µm. All values are within 0.5 pixel size, which indicates that the designed spectrometer exhibits high spectral performance.

## 4. Results

### 4.1. Fabrication of the Spectrometer

#### 4.1.1. Fabrication of the Immersed Gratings

**Figure 19:**
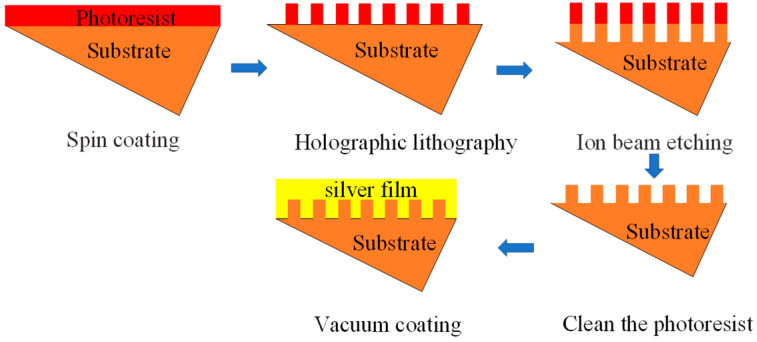
Fabrication process flow of the immersed grating.

The immersed gratings used in this study are fabricated using a holographic lithography–ion-beam etching process [41]. The process flow is shown in Figure 19 and mainly consists of five steps: photoresist coating, holographic lithography, ion beam etching, photoresist removal and cleaning, and reflective film coating.

The maximum size of the immersed grating employed in the spectrometer is 290 mm × 150 mm, with a groove density of 2600 lines/mm. For an immersed grating with such a large aperture and high groove density, the key fabrication challenges lie in patterning the grating on a large-aperture quartz prism and maintaining groove uniformity across the entire grating surface. This groove uniformity directly affects the wavefront error and diffraction efficiency of the immersed grating. To address these challenges, several control measures are implemented in the key fabrication steps. First, during photoresist coating, the thickness uniformity of the photoresist on the large-aperture prism surface is strictly controlled, with the thickness deviation maintained within a tightly controlled range. Second, during holographic lithography, high illumination uniformity of the interference fringes over a 400 mm exposure aperture is achieved by optimizing the optical exposure setup and adjusting the exposure dose and development time. Finally, the photoresist grating pattern is transferred into the quartz substrate by Ar ion-beam etching, after which a dense metallic reflective coating is deposited on the grating surface.

**Figure 20:**
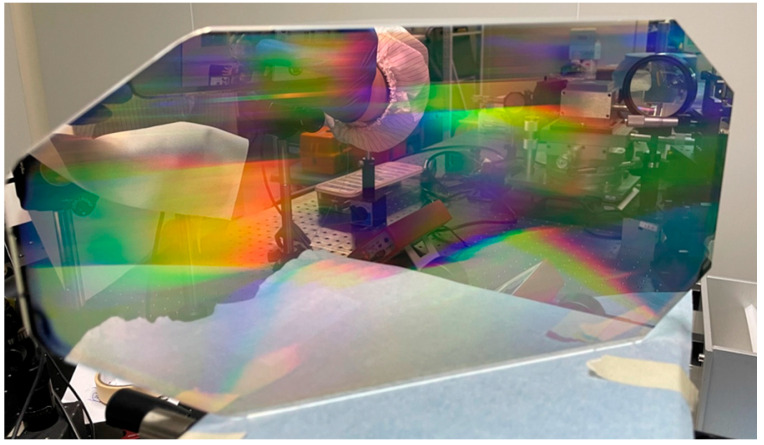
The fabricated fused silica immersed grating of B2 channel. Dimensions: 248 mm × 173 mm × 138.5 mm; groove density: 1434 lines/mm.

Figure 20 shows the fused silica immersed grating for the B2 channel (other channels’ immersed grating are similar, and B2 is shown as a representative), with dimensions of approximately 248 mm × 173 mm × 138.5 mm and a groove density of 1434 lines/mm.

**Figure 21:**
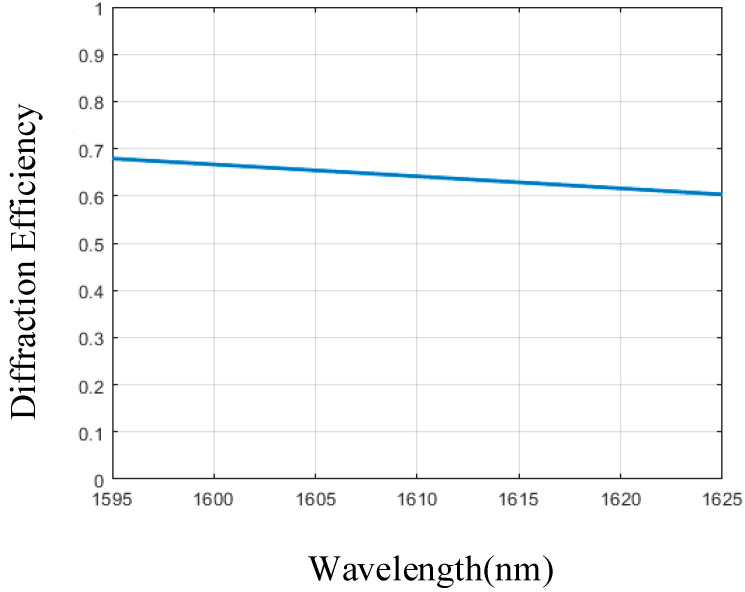
Diffraction-efficiency measurement results of the B2-channel immersed grating.

The diffraction-efficiency measurement results of the B2-channel immersed grating are shown in Figure 21. The measured diffraction efficiency within the working band is above 60%, which is slightly lower than the theoretical value. The deviation is mainly attributed to deviations in the actual groove profile from the ideal trapezoidal profile and to non-ideal silver-film deposition.

#### 4.1.2. Spectrometer Alignment

**Figure 22:**
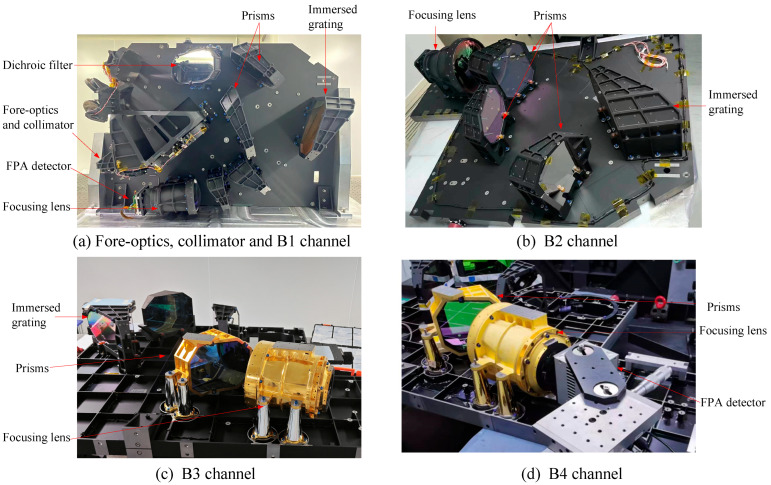
Aligned four-channel immersed grating spectrometer. (**a**) fore-optics, collimator and B1 channel, (**b**) B2 channel, (**c**) B3 channel, (**d**) B4 channel.

The aligned four-channel immersed grating spectrometer is shown in Figure 22. Figure 22a displays the aligned prototypes of the fore-optics, collimator, and B1 channel. Figure 22b–d displays the aligned prototypes of the B2 to B4 channels, respectively.

### 4.2. Test of Spectrometer

**Figure 23:**
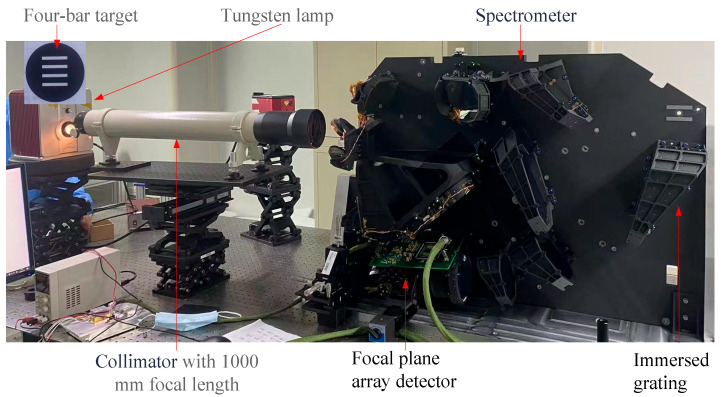
The MTF measurement system.

**Figure 24:**
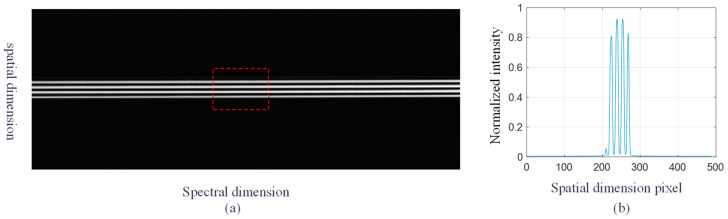
(**a**) The four-bar target image; (**b**) its normalized intensity along the spatial dimension.

Following the fabrication of the spectrometer, a series of measurements is conducted to evaluate the optical performance of the spectrometer. MTF is used to evaluate the imaging performance of the spectrometer. The MTF measurement system is shown in Figure 23. The measurement system consists of a tungsten lamp that generates a continuous spectrum, a collimator with 1000 mm focal length, the fabricated spectrometer, and a focal plane array detector. During the measurement, a four-bar target illuminated by a tungsten lamp is positioned at the focal plane of the collimator. The collimated light is then directed into the spectrometer, and an image of the target is captured by the focal plane array detector, as shown in Figure 24a. The distribution of the normalized intensity of the image along the spatial dimension is shown in Figure 24b. The MTF of the spectrometer is derived by calculating the contrast transfer function of this four-bar target image. Table 6 presents the MTF of the central wavelengths of each channel. The average MTF at the Nyquist frequency corresponding to 10-pixel binning in the spatial dimension is higher than 0.71.

Compared with the design value, the measured MTF decreases by approximately 15%. The reasons are as follows: Firstly, the polarization scrambler reduced the MTF of the spectrometer. The scrambler is used to reduce the polarization sensitivity of the spectrometer to meet the retrieval accuracy requirement. It introduces a wavefront error that broadens the point spread function, thereby reducing the MTF. Secondly, due to the influence of tolerances such as processing and alignment, the MTF of the system decreases by 10% after tolerance analysis. Additionally, the measured MTF includes the MTF of the collimator, which also leads to a decrease in the tested value.

The spectral performance evaluation of the spectrometer includes spectral resolution, smile, and keystone. The spectral measurement setup is shown in Figure 22. Monochromatic light is provided by a tunable external cavity diode laser (TEC-500-1650-020, Sacher Lasertechnik, Marburg, Germany), which operates over a wavelength range of 1595–1750 nm with a typical output power of 15 mW and a linewidth of <1 MHz. The laser beam is homogenized by an integrating sphere (Labsphere, North Sutton, NH, USA) coated with Spectraflect high-reflectance material. The sphere has a diameter of 150 mm and a 50 mm output port, achieving an irradiance uniformity >98% at 1.6 μm. After passing through the integrating sphere, the homogenized light enters the entrance pupil of the spectrometer and is finally imaged onto a custom-built InGaAs focal plane array detector. This detector was developed by the Shanghai Institute of Technical Physics (SITP), Chinese Academy of Sciences, and features a pixel size of 25 μm, an array format of 488 × 1304 pixels, and thermoelectric cooling.

**Figure 25:**
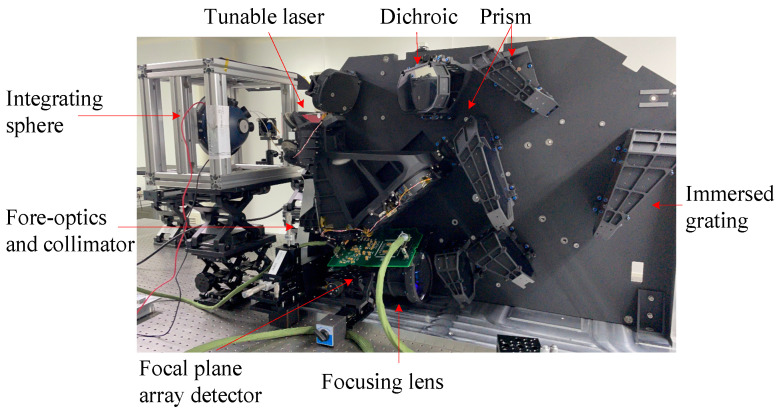
Spectral measurement setup.

**Figure 26:**
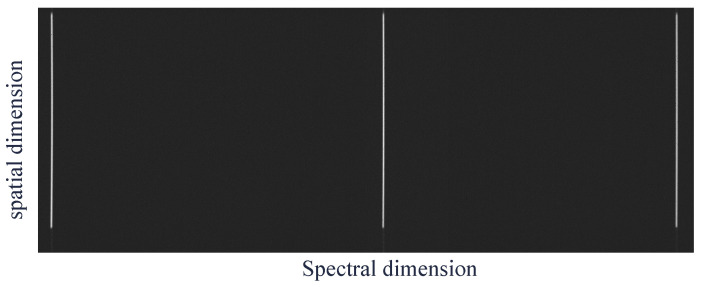
Spectrum lines of the B2 channel.

**Figure 27:**
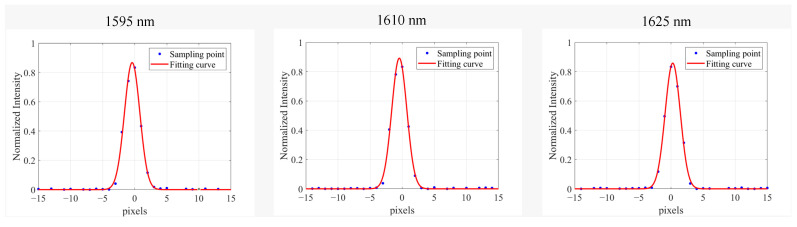
The spectral response function of the B2 channel.

The spectral measurement setup is shown in Figure 25. A tunable laser is used to provide monochromatic light, which can be adjusted within a continuous spectral range. The monochromatic light is first homogenized by an integrating sphere and then illuminates the entrance pupil of the spectrometer. After passing through the spectrometer, the light is ultimately imaged on the focal plane array detector. Figure 26 shows three spectral lines sequentially captured by the focal plane array detector of the B2 channel, corresponding to wavelengths of 1.595 µm, 1.610 µm, and 1.625 µm. The intensity distribution of each spectral line along the dispersion direction was fitted with a Gaussian function. The fitting results for the three lines are presented in Figure 27. The spectral resolution is determined by calculating the full width at half maximum (FWHM) of the Gaussian curve and the linear dispersion.

**Figure 28:**
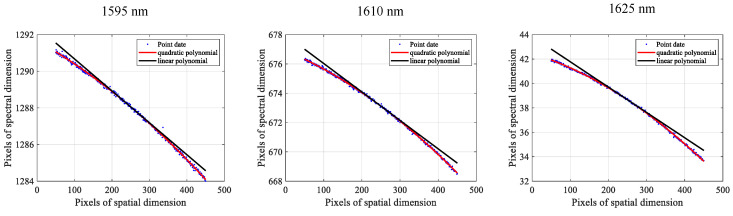
Smile of the B2 channel spectral lines.

The smile is quantified by fitting the centroid curve of the spectral lines. The centroid positions were fitted using both a quadratic polynomial and a linear polynomial. The fitting results for the three spectral lines are presented in Figure 28. The value of a smile is the maximum difference between the quadratic curve and the straight line in the spectral dimension direction. This calculation method eliminates the influence of detector tilt.

**Figure 29:**
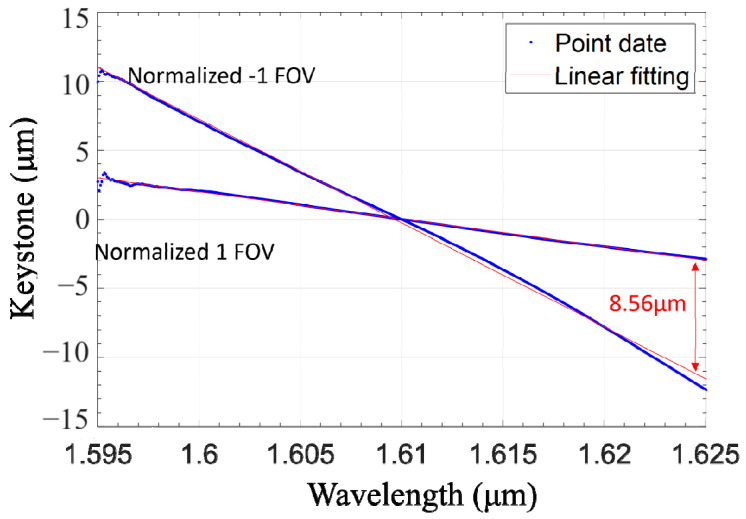
Keystone of the B2 channel.

Keystone is measured using a continuous-spectrum tungsten lamp source, rather than a tunable laser. Keystone refers to the difference in the height of slit images at different wavelengths [42]. By fitting the image points of the edge FOVs, two straight lines can be obtained. The Keystone value can be calculated by the difference in slopes of two lines and the discrete width. Figure 29 shows the fitting lines of the edge FOVs of the B2 channel, and its keystone is 8.56 µm.

The spectral resolution measurement results for the four channels of the spectrometer are presented in Table 7 and the smile and keystone results are presented in Table 8.

The spectral resolution of B1 to B4 is better than 0.04 nm, 0.07 nm, 0.09 nm, and 0.1 nm, respectively. The maximum smile is 22.26 µm, which is less than one pixel. The maximum keystone is 8.56 µm, which is less than 0.5 pixels.

The tested spectral resolutions exhibit deviations from the designed values. The sources are as follows: Firstly, the slit width used in the measurement is slightly smaller than the designed value. Secondly, the Gaussian function fitting error caused by the small number of sampling points leads to the tested values being smaller. The tested smile and keystone values exceed the designed values, primarily due to alignment tolerances of the optical components. Among these factors, the alignment of the immersed grating has the most significant influence on the smile. As detailed in Table 9, a misalignment of 1′ around the y-axis increases the smile by 12.27 µm, which is far more critical than rotations around the x-axis or z-axis.

Overall, although some deviations exist between the tested performance and the designed values, the experimental results still demonstrate the engineering feasibility of the proposed spectrometer. The observed deviations mainly arise from fabrication and alignment tolerances, which lead to reduced MTF and increased smile and keystone relative to the designed values. Nevertheless, the tested spectral performance remains within acceptable engineering limits, supporting the practical applicability of the proposed design for greenhouse-gas-monitoring spectrometers.

## 5. Discussion

The optical system of the spectrometer designed and fabricated in this work achieves a wide swath and high spatial resolution. Nevertheless, certain limitations remain. First, the fore-optics and collimator are based on even-order aspheric mirrors. While this configuration meets the required imaging quality, the system volume could be further reduced. Second, all four spectral channels employ fused-silica immersed gratings. Although fused-silica gratings offer high angular dispersion, additional prisms are necessary to correct the induced smile and anamorphic beam compression, which increases alignment complexity, volume, and weight.

Future greenhouse gas monitoring missions require spectrometers with a wider swath to improve detection efficiency, while also demanding a reduction in volume to lower launch costs. To overcome these limitations, freeform optical elements are introduced into the fore-optics and collimator. Freeform surfaces can correct high-order aberrations over a large field of view while significantly reducing the overall system volume. For the core dispersive element, silicon immersed gratings are adopted to replace the fused-silica immersed gratings in the B2–B4 channels (1.6–2.3 μm band, where silicon exhibits high transmittance). With a refractive index of approximately 3.5, silicon provides much higher angular dispersion, thereby reducing the grating size and the overall spectrometer volume while maintaining or even improving the spectral resolution. Moreover, the higher refractive index reduces the diffraction angle inside the grating, and through parameter optimization it is possible to achieve negligible smile and anamorphic beam compression without the need for additional correcting prisms. This is beneficial for simplifying the optical system and reducing both its volume and weight. However, it should be noted that the fabrication of silicon immersed gratings remains challenging and requires further advances in high-precision etching and coating processes.

## 6. Conclusions

In this paper, the optical design and fabrication of a spectrometer for global and regional GHG monitoring are presented. The system achieves a 100 km swath width, a spatial resolution of 3 km × 3 km, and high spectral resolution. It consists of four channels centered at 0.76 μm (O_2_-A band), 1.61 μm (weak CO_2_ band), 2.06 μm (strong CO_2_ band), and 2.30 μm (CH_4_ band), with corresponding spectral resolutions of 0.04 nm, 0.07 nm, 0.09 nm, and 0.10 nm, respectively. The four channels share a common slit, which reduces system volume and inter-channel spatial registration errors. To meet the performance requirements of each channel, the spectral resolution and F-number are first determined through radiative-transfer modeling and signal-to-noise-ratio analysis, followed by the optical design of the system. For the dispersion module, a prism-based simultaneous correction method is developed to correct the smile and anamorphic beam compression induced by high-dispersion immersed gratings. Combined with an analytical approach, this method enables accurate determination of the initial parameters of the dispersion module and reduces trial-and-error in parameter selection. Based on the completed design, the core spectral components and the full spectrometer are fabricated. In particular, the large-sized immersed gratings with high groove density are manufactured using holographic lithography–ion-beam etching, achieving an average diffraction efficiency of 65% over the operating wavelength range. The spectrometer is then aligned and tested. The results show that the developed spectrometer satisfies the specified requirements for imaging quality and spectral resolution, with both smile and keystone controlled to within one pixel. These results demonstrate that the proposed system provides a high-performance technical solution for next-generation spaceborne GHG monitoring by effectively integrating high spectral resolution, wide-swath coverage, and high spatial resolution. The proposed design is expected to support regional emission-source analysis and improve observation efficiency for global carbon-cycle studies.

## Figures and Tables

**Table 1:** Parameters used in the simulation.

Atmospheric Profile Model	1976 US Standard Atmosphere
Spectral range	1.595 µm–1.625 µm
CO_2_ concentration	400 ppm
Surface Albedo	0.05
Observation method	Nadir Point Observation
Solar zenith angle	60°
Slit function type	Gaussian

**Table 2:** The required SNR at different spectral resolutions.

Spectral Resolution (nm)	Relative Radiance Change	Minimum Required SNR (Covering Single Absorption Line)	Minimum Required SNR (Covering 31 Absorption Lines)
0.05	13.39 × 10^−4^	747	167
0.07	9.92 × 10^−4^	1008	181
0.15	4.98 × 10^−4^	2008	449
0.20	3.80 × 10^−4^	2631	588

**Table 3:** Specifications and parameters of the spectrometer.

Specifications and Parameters	Parameter Values			
Orbital altitude/km	836			
Swath width/km	100			
Field of view (FOV)/°	7			
Spatial resolution/km	3			
Spectral channel	B1 (0.76 µm band)	B2 (1.61 µm band)	B3 (2.06 µm band)	B4 (2.3 µm band)
Wavelength range/μm	0.7525–0.7675	1.595–1.625	2.04–2.08	2.275–2.325
Spectral resolution/nm	0.04	0.07	0.09	0.10
F-number	1.70	1.80	1.73	1.50
Focal length/mm	75	81	77	67

**Table 4:** Parameters of grating spectrometer for GHG monitoring.

	Status	Dispersion Element	Swath Width (km)	Spatial Resolution (km)	Wavelength Range (μm)	Spectral Resolution (nm)
TROPOMI [14,15]	launched in 2017	Immersed grating	2600	7 × 7	0.27–0.49 0.710–0.775 2.31–2.39	0.45–0.65 0.35–0.45 0.225
ACGS [1,12]	launched in 2016	plane gratings	18	1 × 2	0.758~0.778 1.594~1.624 2.04~2.08	0.04 0.08 0.1
OCO-2 [10,11]	launched in 2014	plane gratings	10.6	1.29 × 2.25	0.757~0.772 1.59~1.62 2.04~2.08	0.04 0.08 0.1
GOSAT-GW [22,23]	launched in 2025	plane gratings	90	3 × 3	0.45–0.49 0.747–0.783 1.590–1.654	0.5 0.05 0.2
CO2M [25,26]	under-development	Immersed grating	250	2 × 2	0.747–0.773 1.590–1.675 1.990–2.095	0.12–0.35

**Table 5:** Smile and keystone for all channels and wavelengths.

	Wavelength (nm)	Smile (µm)	Keystone (µm)
B1	752.5	8.1	3.6
	760	0.7	
	767.5	7.6	
B2	1595	4.9	4.9
	1610	0.9	
	1625	3.6	
B3	2040	2.0	0.3
	2060	0.8	
	2080	2.9	
B4	2275	2.9	0.2
	2300	0.6	
	2325	3.1	

**Table 6:** The MTF of the four channels of the spectrometer.

	Wavelength (nm)	MTF @ 2 Lines/mm				
		Normalized Field of View			Average	Standard Deviation
		−1	0	1		
B1	757.5	0.7709	0.7704	0.7773	0.7729	0.0038
	765	0.7723	0.7683	0.7738	0.7715	0.0029
	772.5	0.7741	0.7722	0.7775	0.7746	0.0027
B2	1595	0.7557	0.7586	0.7621	0.7588	0.0032
	1610	0.7599	0.7617	0.7646	0.7621	0.0024
	1625	0.7605	0.7607	0.7646	0.7619	0.0023
B3	2040	0.7162	0.7167	0.6984	0.7104	0.0104
	2060	0.7235	0.7256	0.6965	0.7152	0.0156
	2080	0.7417	0.7383	0.6906	0.7235	0.0279
B4	2275	0.7672	0.7689	0.7614	0.7658	0.0040
	2300	0.7573	0.7751	0.7567	0.7630	0.0103
	2325	0.7687	0.7503	0.7304	0.7498	0.0192

**Table 7:** The four-channel spectral resolution of the spectrometer.

Channel	Wavelength (nm)	Spectral Resolution (nm)				
		Normalized Field of View			Average	Standard Deviation
		−1	0	1		
B1	752.5	0.0364	0.0362	0.0347	0.0358	0.00093
	760	0.0342	0.0352	0.0343	0.0356	0.00055
	767.5	0.0364	0.0370	0.0352	0.0362	0.00090
B2	1595	0.0632	0.0597	0.0572	0.0600	0.00301
	1610	0.0633	0.0611	0.0606	0.0617	0.00145
	1625	0.0640	0.0636	0.0630	0.0635	0.00050
B3	2040	0.0744	0.0723	0.0720	0.0729	0.00129
	2060	0.0710	0.0747	0.0737	0.0731	0.00193
	2080	0.0786	0.0798	0.0786	0.0790	0.00069
B4	2275	0.0952	0.0926	0.0920	0.0933	0.00170
	2300	0.0857	0.0830	0.0805	0.0831	0.00261
	2325	0.0895	0.0932	0.0901	0.091	0.00193

**Table 8:** The four-channel smile and keystone of the spectrometer.

Channel	Wavelength (nm)	Smile (μm)	Keystone (μm)
B1	752.5	13.00	5.70
	760	5.24	
	767.5	4.67	
B2	1595	11.29	8.56
	1610	15.60	
	1625	22.26	
B3	2040	8.12	6.34
	2060	5.95	
	2080	4.63	
B4	2275	1.39	5.28
	2300	3.17	
	2325	3.31	

**Table 9:** The smile variation is caused by the alignment tolerance of the immersed grating.

	Rotation of Immersed Grating by 1′		
	X	Y	Z
Wavelength (nm)	smile variation(µm)		
1590	2.23	11.55	−4.33
1610	0.07	11.66	−4.33
1625	2.60	12.27	−4.55

## Data Availability

The data presented in this study are available on request from the corresponding author due to intellectual property protection and privacy.
